# CeRebrUm and CardIac Protection with ALlopurinol in Neonates with Critical Congenital Heart Disease Requiring Cardiac Surgery with Cardiopulmonary Bypass (CRUCIAL): study protocol of a phase III, randomized, quadruple-blinded, placebo-controlled, Dutch multicenter trial

**DOI:** 10.1186/s13063-022-06098-y

**Published:** 2022-02-23

**Authors:** Raymond Stegeman, Maaike Nijman, Johannes M. P. J. Breur, Floris Groenendaal, Felix Haas, Jan B. Derks, Joppe Nijman, Ingrid M. van Beynum, Yannick J. H. J. Taverne, Ad J. J. C. Bogers, Willem A. Helbing, Willem P. de Boode, Arend F. Bos, Rolf M. F. Berger, Ryan E. Accord, Kit C. B. Roes, G. Ardine de Wit, Nicolaas J. G. Jansen, Manon J. N. L. Benders, on behalf of the CRUCIAL trial consortium

**Affiliations:** 1 Department of Neonatology, Wilhelmina Children’s Hospital, University Medical Center (UMC) Utrecht, Utrecht University, KE 04.123.1, PO Box 85909, 3508 AB Utrecht, The Netherlands; 2 Department of Pediatric Cardiology, Wilhelmina Children’s Hospital, UMC Utrecht, Utrecht University, Utrecht, The Netherlands; 3 Department of Pediatric Intensive Care, Wilhelmina Children’s Hospital, UMC Utrecht, Utrecht University, Utrecht, The Netherlands; 4 Congenital Cardiothoracic Surgery, Wilhelmina Children’s Hospital, UMC Utrecht, Utrecht University, Utrecht, The Netherlands; 5 Utrecht Brain Center, UMC Utrecht, Utrecht University, Utrecht, The Netherlands; 6 Department of Obstetrics, Wilhelmina Children’s Hospital, UMC Utrecht, Utrecht University, Utrecht, The Netherlands; 7 Department of Pediatrics, Division of Pediatric Cardiology, Academic Center for Congenital Heart Disease, Erasmus Medical Center (MC) - Sophia Children’s Hospital, Rotterdam, The Netherlands; 8 Department of Cardiothoracic Surgery, Erasmus MC, Erasmus University Rotterdam, Rotterdam, The Netherlands; 9 Department of Pediatrics, Division of Pediatric Cardiology, Academic Center for Congenital Heart Disease, Radboudumc - Amalia Children’s Hospital, Nijmegen, The Netherlands; 10 Department of Neonatology, Radboudumc, Radboud Institute for Health Sciences, Amalia Children’s Hospital, Nijmegen, The Netherlands; 11 Division of Neonatology, Beatrix Children’s Hospital, UMC Groningen, University of Groningen, Groningen, The Netherlands; 12 Center for Congenital Heart Diseases, Pediatric Cardiology, Beatrix Children’s Hospital, UMC Groningen, University of Groningen, Groningen, The Netherlands; 13 Center for Congenital Heart Diseases, Department of Cardiothoracic Surgery, UMC Groningen, University of Groningen, Groningen, The Netherlands; 14 Department of Health Evidence, Section Biostatistics, Radboudumc, Radboud University Nijmegen, Nijmegen, The Netherlands; 15 Julius Center for Health Sciences and Primary Care, UMC Utrecht, Utrecht University, Utrecht, The Netherlands; 16 Department of Pediatrics, Beatrix Children’s Hospital, UMC Groningen, University of Groningen, Groningen, The Netherlands

**Keywords:** Neonate, Congenital heart disease, Allopurinol, Brain injury, Cardiac function, Neurodevelopmental outcome

## Abstract

**Background:**

Neonates with critical congenital heart disease (CCHD) undergoing cardiac surgery with cardiopulmonary bypass (CPB) are at risk of brain injury that may result in adverse neurodevelopment. To date, no therapy is available to improve long-term neurodevelopmental outcomes of CCHD neonates. Allopurinol, a xanthine oxidase inhibitor, prevents the formation of reactive oxygen and nitrogen species, thereby limiting cell damage during reperfusion and reoxygenation to the brain and heart. Animal and neonatal studies suggest that allopurinol reduces hypoxic-ischemic brain injury and is cardioprotective and safe. This trial aims to test the hypothesis that allopurinol administration in CCHD neonates will result in a 20% reduction in moderate to severe ischemic and hemorrhagic brain injury.

**Methods:**

This is a phase III, randomized, quadruple-blinded, placebo-controlled, multicenter trial. Neonates with a prenatal or postnatal CCHD diagnosis requiring cardiac surgery with CPB in the first 4 weeks after birth are eligible to participate. Allopurinol or mannitol-placebo will be administered intravenously in 2 doses early postnatally in neonates diagnosed antenatally and 3 doses perioperatively of 20 mg/kg each in all neonates. The primary outcome is a composite endpoint of moderate/severe ischemic or hemorrhagic brain injury on early postoperative MRI, being too unstable for postoperative MRI, or mortality within 1 month following CPB. A total of 236 patients (*n* = 188 with prenatal diagnosis) is required to demonstrate a reduction of the primary outcome incidence by 20% in the prenatal group and by 9% in the postnatal group (power 80%; overall type 1 error controlled at 5%, two-sided), including 1 interim analysis at *n* = 118 (*n* = 94 with prenatal diagnosis) with the option to stop early for efficacy. Secondary outcomes include preoperative and postoperative brain injury severity, white matter injury volume (MRI), and cardiac function (echocardiography); postnatal and postoperative seizure activity (aEEG) and regional cerebral oxygen saturation (NIRS); neurodevelopment at 3 months (general movements); motor, cognitive, and language development and quality of life at 24 months; and safety and cost-effectiveness of allopurinol.

**Discussion:**

This trial will investigate whether allopurinol administered directly after birth and around cardiac surgery reduces moderate/severe ischemic and hemorrhagic brain injury and improves cardiac function and neurodevelopmental outcome in CCHD neonates.

**Trial registration:**

EudraCT 2017-004596-31. Registered on November 14, 2017. ClinicalTrials.gov NCT04217421. Registered on January 3, 2020

**Supplementary Information:**

The online version contains supplementary material available at 10.1186/s13063-022-06098-y.

## Administrative information

**Table:** 

Title	CeRebrUm and CardIac Protection with ALlopurinol in Neonates with Critical Congenital Heart Disease Requiring Cardiac Surgery with Cardiopulmonary Bypass (CRUCIAL): study protocol of a phase III, randomized, quadruple-blinded, placebo-controlled, Dutch multicenter trial
Trial registration	EudraCT (clinicaltrialsregister.eu) 2017-004596-31, registered November 14, 2017. ClinicalTrials.gov NCT04217421, registered January 3, 2020. All items from the World Health Organization Trial Registration Data Set are included within the protocol.
Protocol version	February 2, 2020, protocol version 2.1.
Funding	This study is funded by ZonMw, as part of the Goed Geneesmiddelen Gebruik, Grote Trials Ronde II program (https://www.zonmw.nl/en/ project number 848042002), the Hartekind foundation (https://www.hartekind.nl), and Friends of the Wilhelmina Children’s Hospital foundation (https://vriendenumcutrecht-wkz.nl).
Author details	Raymond Stegeman^1-5^*, Maaike Nijman^1,2,5^*, Johannes M.P.J. Breur^2^, Floris Groenendaal^1,5^, Felix Haas^4^, Jan B. Derks^6^, Joppe Nijman^3^, Ingrid M. van Beynum^7^, Yannick J.H.J. Taverne^8^, Ad J.J.C. Bogers^8^, Willem A. Helbing^7,9^, Willem P. de Boode^10^, Arend F. Bos^11^, Rolf M.F. Berger^12^, Ryan E. Accord^13^, Kit C.B. Roes^14^, G. Ardine de Wit^15^, Nicolaas J.G. Jansen^3,16^**, Manon J.N.L. Benders^1,5^**, on behalf of the CRUCIAL trial consortium*** *Both authors share first authorship; **Both authors share last authorship; ***Contributors: -ACAHA Erasmus MC Rotterdam/Radboudumc Nijmegen: Koen F. M. Joosten, Pieter C. van de Woestijne, Inge I. de Liefde, Antony van Dijk, Dafni Charisopoulou, Sinno H. P. Simons, Robin van der Lee, Jérôme M. J. Cornette, Neeltje E. M. van Haren; -UMC Groningen: Sara C. Arrigoni, Leonie K. Duin, Martin C.J. Kneyber, Elisabeth M.W. Kooi , Joost M.A.A. van der Maaten, Linda C. Meiners, Mirthe J. Mebius, and Gideon J. du Marchie Sarvaas; -UMC Utrecht: Nathalie H.P. Claessens, Bram van Wijk, Paul H. Schoof, Trinette J. Steenhuis, Henriette ter Heide, Roel de Heus, Mireille N. Bekker, Roelie M. Wösten-van Asperen, Erik Koomen, Kim van Loon, and Nicole van Belle-van Haaren; -Biostatistics Julius Center: Stavros Nikolakopoulos, Rene Eijkemans, Daniela Cianci; -Central Pharmacy: Arief Lalmohamed, Karin Rademaker. ^1^Department of Neonatology, ^2^Pediatric Cardiology, ^3^Pediatric Intensive Care, ^4^Congenital Cardiothoracic Surgery, Wilhelmina Children’s Hospital, University Medical Center (UMC) Utrecht, Utrecht University, Utrecht, The Netherlands; ^5^Utrecht Brain Center, UMC Utrecht, Utrecht University, Utrecht, The Netherlands; _6_Department of Obstetrics, Wilhelmina Children’s Hospital, UMC Utrecht, Utrecht University, Utrecht, The Netherlands; ^7^Department of Pediatrics, Division of Pediatric Cardiology, Academic Center for Congenital Heart Disease, Erasmus Medical Center (MC) - Sophia Children’s Hospital, Rotterdam, The Netherlands; ^8^Department of Cardiothoracic Surgery, Erasmus MC, Erasmus University Rotterdam, Rotterdam, The Netherlands; ^9^Department of Pediatrics, Division of Pediatric Cardiology, Academic Center for Congenital Heart Disease, Radboudumc - Amalia Children’s Hospital, Nijmegen, The Netherlands; ^10^Department of Neonatology, Radboudumc, Radboud Institute for Health Sciences, Amalia Children’s Hospital, Nijmegen, The Netherlands; ^11^Division of Neonatology, Beatrix Children’s Hospital, UMC Groningen, University of Groningen, Groningen, The Netherlands; _12_Center for Congenital Heart Diseases, Pediatric Cardiology, Beatrix Children’s Hospital, UMC Groningen, University of Groningen, Groningen, The Netherlands; ^13^Center for Congenital Heart Diseases, Department of Cardiothoracic Surgery, UMC Groningen, University of Groningen, Groningen, The Netherlands; ^14^Department of Health Evidence, Section Biostatistics, Radboudumc, Radboud University Nijmegen, Nijmegen, The Netherlands; ^15^Julius Center for Health Sciences and Primary Care, UMC Utrecht, Utrecht University, Utrecht, The Netherlands; ^16^Department of Pediatrics, Beatrix Children’s Hospital, UMC Groningen, University of Groningen, Groningen, The Netherlands.
Name and contact information for the trial sponsor	S. Veersema, MD, PhD. Medical Research Manager, Division Woman and Baby. UMC Utrecht, Wilhelmina Children’s Hospital, KE04.123.1, P.O. Box 85090, 3508 AB Utrecht, The Netherlands. Email: s.veersema@umcutrecht.nl
Role of sponsor	The subsidizing parties will not play a role in the design of the study, data collection, management, analyses and interpretation of the data, writing of the manuscript, or decision to submit the report for publication. The study sponsor (UMC Utrecht) will have ultimate authority over these activities.

## Background

Congenital heart disease (CHD) is the most common congenital anomaly in newborns. Critical CHD (CCHD) has an incidence of 3 per 1000 live births and concerns severely ill neonates that require cardiac surgery with cardiopulmonary bypass (CPB) shortly after birth [1]. CCHD includes univentricular heart physiology like hypoplastic left and right heart syndrome; transposition of the great arteries; aortic arch anomalies such as coarctation, interruption, and hypoplasia; truncus arteriosus; total anomalous pulmonary venous connection; and tetralogy of Fallot [1]. Today, 80–90% of CCHD neonates survive until adulthood due to improvements in surgical procedures and perioperative care [2]. Nevertheless, enhancing the quality of life in children growing up with CCHD remains challenging due to long-term neurodevelopmental sequelae, such as cognitive, behavioral, and motor impairments. These sequelae have a financial impact on society, and CCHD is associated with a high number of disability-adjusted life years [3, 4].

Delayed brain development starts already in utero, persists into the neonatal period, and is the result of the underlying cardiovascular defect causing altered cerebral perfusion and reduced oxygenation [5–7]. The underdeveloped brain of CCHD neonates is at high risk for injury early after birth and around cardiac surgery with CPB. White matter injury and strokes with gray matter involvement are commonly observed on magnetic resonance imaging (MRI) and are thought to be the result of hypoxic-ischemic or thromboembolic events [5]. Preoperatively, up to 30% of the CCHD neonates show ischemic brain lesions, while additional injury after cardiac surgery is found in 51% [8].

The cascade to ischemic brain lesions starts with hypoxia, which leads to cerebral energy depletion, excitotoxicity, increased cell influx of calcium, apoptosis, increase in hypoxanthine, and production of pro-radicals [9–11]. After reperfusion and reoxygenation, the enzyme xanthine oxidase (XO) catalyzes the production of superoxide, which normally only accounts for a small part of the produced reactive oxygen species (ROS) [12]. During reperfusion, large quantities of XO are released into the circulation, possibly reacting with plasma purine substrates and molecular oxygen to produce ROS and reactive nitrogen species (RNS), resulting in a state of redox imbalance. This activates an inflammatory response with the formation of pro- and anti-inflammatory cytokines [11, 13, 14]. The development of neuroprotective drugs targeting specific steps in this cascade of events might have the potential to limit hypoxic-ischemic brain lesions [14].

Several perioperative neuroprotective approaches have been explored in the CCHD population. Prior investigated interventions focused on tight glycemic control, alpha-stat versus pH-stat blood gas management, maintenance of high hematocrit levels, cooling, and perfusion techniques such as deep hypothermic circulatory arrest, low flow CPB, and regional cerebral perfusion [15]. Solely maintaining a hematocrit level above 24% during CPB appears to be favorable for the outcome [16–18]. With regard to pharmacological neuroprotection, allopurinol seems the most promising drug in CCHD neonates [19]. Allopurinol, a XO inhibitor, reduces the production of ROS and RNS during reperfusion and reoxygenation [20]. In addition, allopurinol preserves cerebral energy metabolism, is a pro-radical chelator of non-protein bound iron, and has anti-inflammatory properties [21–24].

Prior animal and small neonatal studies suggest neuro- and cardioprotective effects of allopurinol [25]. Clancy et al. showed that the use of perioperative allopurinol resulted in a lower composite rate of clinical seizures, cardiac events, coma, and death in infants with hypoplastic left heart syndrome [26]. During intracardiac repair for tetralogy of Fallot, allopurinol had beneficial effects on the use of inotropes, duration of mechanical ventilation, and intensive care stay [24]. Postnatal administration of allopurinol improved neurodevelopmental outcomes in infants with moderate hypoxic-ischemic encephalopathy [27–29]. Neuronal injury, as measured by S100B and neuroketal levels, was reduced after antenatal administration of allopurinol to fetuses suspected of hypoxia, especially in females. No severe side effects of allopurinol were observed in all previously performed neonatal studies, and neither in the currently ongoing ALBINO Study (NCT03162653) [24, 26–33]. Only in 4.5% of mothers receiving allopurinol, reversible irritation of perivascular tissue was reported after intravenous administration [30].

Previous neuroprotective research in neonatal CCHD focused mainly on interventions exclusively in the perioperative period. However, brain injury occurs already preoperatively [8, 34, 35]. Pretreatment with neuroprotective drugs as allopurinol, which blocks the cascade to ischemic brain lesions, should therefore be administered both early after birth and around cardiac surgery with CPB. Additionally, pre- and postoperative brain MRI are needed to evaluate the effects of postnatal and perioperative treatments on brain lesions separately. Perioperative MRI can be used as a “bridging biomarker” for long-term neurodevelopmental outcomes, since it is known that moderate to severe ischemic brain injury is associated with poorer outcomes at school-age [36].

Therefore, this phase III, randomized, quadruple-blinded, placebo-controlled, multicenter trial will evaluate CeRebrUm and CardIac protection with ALlopurinol in neonates with CCHD Requiring Cardiac Surgery with CPB (CRUCIAL). The aim of this study is to reduce moderate/severe ischemic and hemorrhagic brain injury and improve cardiac function and neurodevelopmental outcomes in CCHD children.

## Methods

The Standard Protocol Items: Recommendations for Interventional Trials (SPIRIT) checklist is provided in Additional file 1 [37].

### Trial design

The proposed study is a phase III, randomized, quadruple-blinded, placebo-controlled, multicenter trial for the superiority of allopurinol versus mannitol-placebo. We hypothesize that allopurinol administration in neonates with CCHD will result in a 20% reduction in the primary outcome, which is defined as the presence of moderate to severe ischemic or hemorrhagic brain injury, being clinically too unstable for postoperative MRI, or mortality.

### Study setting

The trial will be conducted in 4 academic centers for CHD in the Netherlands: University Medical Center (UMC) Utrecht (sponsor), UMC Groningen, and Academic Center for Congenital Heart Disease (ACAHA) in Erasmus Medical Center (MC) Rotterdam and Radboudumc Nijmegen.

### Eligibility criteria

#### Inclusion criteria

Neonates with a prenatally or postnatally confirmed diagnosis of CCHD requiring (anticipated) cardiac surgery with CBP within the first 4 weeks of life are eligible for inclusion. This includes neonates with the following cardiac defects: (1) transposition of the great arteries with or without a ventricular septal defect undergoing arterial switch operation with ventricular septal defect closure if needed; (2) univentricular hearts—hypoplastic left and right heart syndrome or variant undergoing Norwood stage I or Sano palliation; (3) aortic arch anomalies—interrupted, hypoplastic aortic arch, and/or coarctation of the aorta with or without intracardiac defects (ventricular/atrial septal defect or (sub)aortic stenosis) who undergo complete biventricular repair and/or aortic arch repair; and (4) other variants—truncus arteriosus, total anomalous pulmonary venous connection, or tetralogy of Fallot. Written informed consent of both parents is required prior to inclusion.

#### Exclusion criteria

Exclusion criteria include (1) inability to enroll the patient before the start of delivery in case of prenatal diagnosis or 24 h before surgery in case of postnatal diagnosis, (2) uncertainty antenatally whether the heart defect requires cardiac surgery with CPB within the first 4 weeks of life, (3) no anticipated need for CPB during cardiac surgery, (4) gestational age below 36 weeks or a birth weight less than 2000 g, and (5) the patient is deemed incurable or there is a decision of “comfort care only.” Neonates with an underlying syndromic disorder, such as DiGeorge or trisomy 21 syndrome, are not excluded beforehand, as this will not affect the incidence of brain lesions in the composite primary outcome.

### Intervention

Allopurinol 100 mg powder for infusion (PFI) or mannitol-placebo 100 mg PFI is reconstituted in 10 ml sterile water for injection (WFI). Each dose of 20 mg/kg, corresponding to a concentration of 2 ml/kg, will be delivered through peripheral or central line infusion over 10 min using a syringe pump. Blinding is secured as mannitol-placebo PFI has an identical appearance as allopurinol PFI, and with a dose of 20 mg/kg, no clinical effects are expected from mannitol-placebo PFI administration [38]. The study medication will be arranged in patient-dedicated boxes, containing 5 vials of PFI and WFI (prenatal group) or 3 vials of PFI and WFI (postnatal group).

#### Prenatal group

**Fig. 1:**
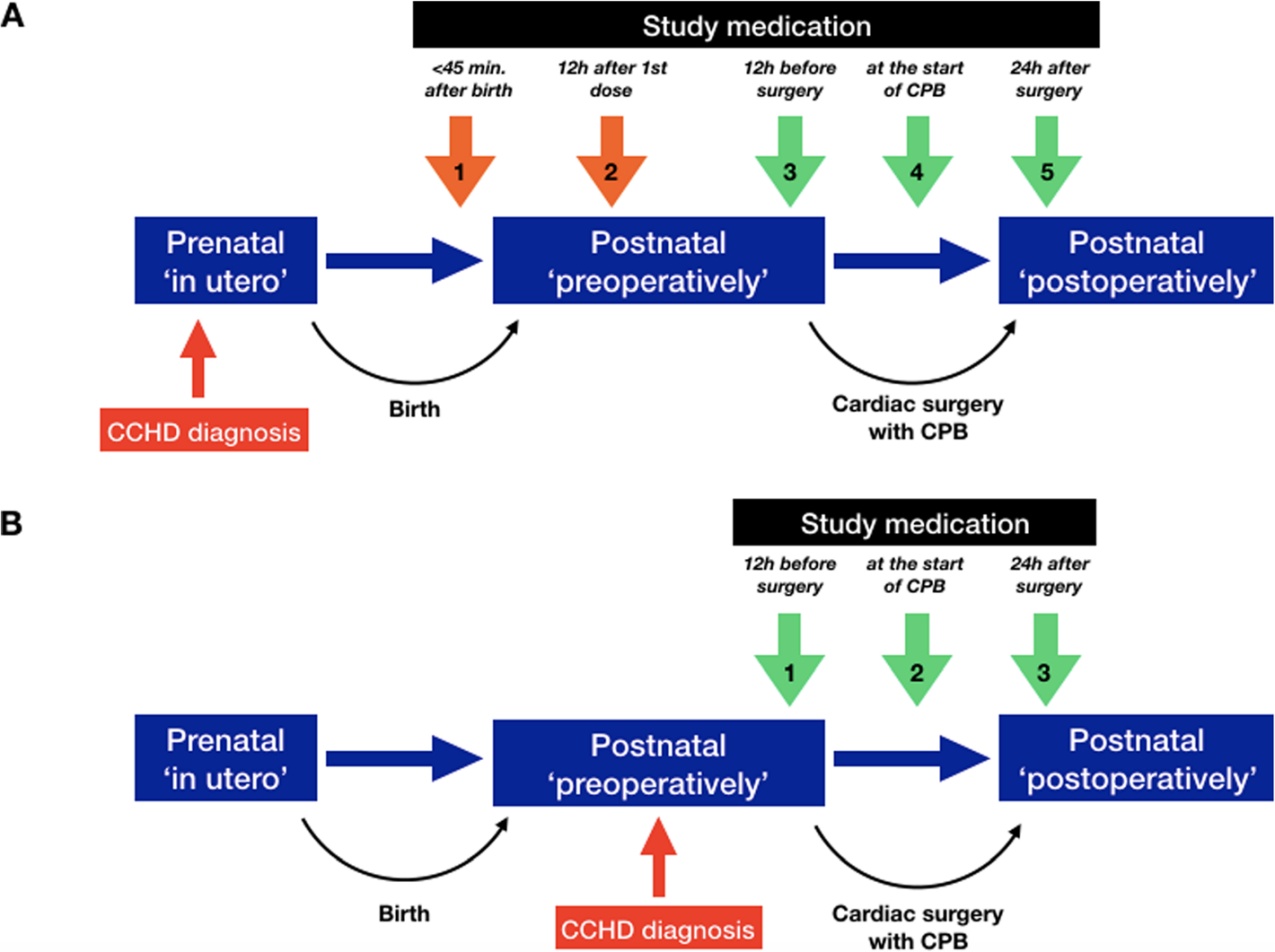
Administration schedule of study medication in both groups. **A** Administration schedule in prenatally diagnosed neonates (prenatal group). **B** Administration schedule in postnatally diagnosed neonates (postnatal group). CCHD, critical congenital heart disease; CPB, cardiopulmonary bypass; h, hours; min, minutes

In neonates that are diagnosed with CCHD before birth, allopurinol PFI or mannitol-placebo PFI 20 mg/kg body weight per administration will be administered early postnatally (within 45 (max. 60) min after birth and 12 h after the first dose), preoperatively (12 h prior to cardiac surgery), intraoperatively (at the start of CPB), and postoperatively (24 h after surgery) (Fig. 1).

#### Postnatal group

Neonates in whom CCHD is diagnosed after birth will only receive the pre-, intra-, and postoperative doses of allopurinol PFI or mannitol-placebo PFI (Fig. 1).

#### Randomization and allocation concealment

Randomization will be performed by Ace Pharmaceuticals in blocking for both the prenatal and postnatal diagnosis group (by Randlist version 1.2 software). Stratification will be conducted per center and for a prenatal diagnosis or postnatal diagnosis of CCHD. The first available box of blinded study medication will be allocated by the study team to consecutive study subjects. Each box will be labeled with a unique medication identification number, which enables identification of treatment allocation for data analyses afterwards or emergency unblinding.

#### Adherence to intervention protocol

To improve adherence to the intervention protocol, the study team will organize multiple training sessions for care providers. After completion of the intervention protocol, every patient-dedicated box will return to the study team. Prior to the destruction of the dispensed study medication, an independent monitor will check the drug accountability records.

#### Criteria for discontinuing or modifying allocated interventions

A physician or local principal investigator may decide to terminate the administration of study medication due to safety concerns. The reason for premature discontinuation will be documented, and the outcome of these participants will be included in the intention-to-treat analysis. Parents are allowed to withdraw the participation of their child from the trial at any time. If consent is withdrawn, no further study procedures will be performed. However, we will ask permission to continue data registration for intention-to-treat analysis.

#### Concomitant treatment

Aside from the trial intervention, patients will be treated according to routine clinical practice in each participating center. Administration of open-label allopurinol will not be allowed.

### Outcomes

The period of primary and secondary outcome assessments for every participant will be a total of 24 months.

#### Primary outcome

The primary composite endpoint, assessed within 1 month after neonatal cardiac surgery with CPB, is defined by the presence of moderate/severe ischemic or hemorrhagic parenchymal brain injury on postoperative MRI or being clinically too unstable for postoperative MRI or mortality. All postoperative MRI scans will be assessed centrally and blinded in the UMC Utrecht by 2 experienced neuro-neonatologists. Moderate brain injury is defined as 4–6 white matter lesions < 2 mm or 1–2 lesions 2–4 mm, single focal infarction of the gray matter > 2 mm, or a single hemorrhage (including cerebellar) 2–5 mm. Severe brain injury is defined as > 6 white matter lesions < 2 mm or > 2 lesions 2–4 mm or lesion(s) > 4 mm, infarction with involvement of both the gray and white matter, multiple infarctions of the gray matter, multiple hemorrhages or a single hemorrhage (including cerebellar) > 5 mm, or involvement of the posterior limb of the internal capsule (motor tracts) or radiatio optica (visual tracts) [8]. The patient is deemed “too unstable for postoperative MRI” when the clinical team considers (transport to) MRI too risky due to hemodynamic instability, e.g., need for inotropic support, (septic) shock, or cardiac arrhythmias.

#### Secondary outcomes

The secondary outcomes that will be compared between the allopurinol and mannitol-placebo groups are presented in Table 1 and include the five elements (domain, specific measurement, specific metric, method of aggregation, and time point) as described by Zarin et al. and Saldanha et al. [55, 56].

**Table 1:** Secondary outcomes

Domain	Specific measurement	Specific metric	Method of aggregation	Time point
Brain injury severity	Brain injury severity score, determined by the assessment of brain injury on MRI in the deep gray matter, white matter, cortex, and cerebellum [39, 40].	Preoperative brain injury severity score, postoperative brain injury severity score, and change between pre- and postoperative brain injury severity score	Mean (and SD) or median (and IQR) per group	Preoperative (4–7 days after birth) Postoperative (5–10 days after surgery or at least within 1 month after surgery)
White matter injury volume	White matter injury volume (mm^3^), determined by manual segmentation with 3D-Slicer or ITK-SNAP 2.0 [41–43].	Preoperative white matter injury volume, postoperative white matter injury volume, and change between pre- and postoperative white matter injury volume	Mean (and SD) or median (and IQR) per group	Preoperative (4–7 days after birth) Postoperative (5–10 days after surgery or at least within 1 month after surgery)
Echocardiographic systolic and diastolic function	Echocardiographic assessment of global ventricular function (normal, mildly reduced, moderately reduced, severely reduced); ejection fraction (%); shortening fraction (%); ventricular dimensions (mm, *Z*-score corrected for BMI); velocity over the cardiac valves, i.e., *V*peak (m/s) and *V*mean (m/s); cardiac valve insufficiency (no, mild, moderate, severe); longitudinal strain (%) and tissue Doppler imaging (myocardial deformation indices (m/s)); and systolic and diastolic pulmonic vein flow (m/s) [44].	Preoperative echocardiographic measurements, postoperative echocardiographic measurements, and change between pre- and postoperative echocardiographic measurements	Distribution per group for categorical variables and mean (and SD) or median (and IQR) per group for continuous variables	Preoperative (4–7 days after birth) Postoperative (5–10 days after surgery or at least within 1 month after surgery)
Seizure activity	Presence or absence of seizure activity on aEEG [45, 46].	Postnatal seizure activity and postoperative seizure activity	Proportion of neonates with seizure activity per group	Postnatal (until at least 24 h after birth) Postoperative (until at least 48 h after surgery)
Regional cerebral oxygen saturation	Regional cerebral oxygen saturation (%), lowest regional cerebral oxygen saturation (%), and duration of regional cerebral oxygen saturation < 45% (minutes) will be assessed by NIRS in intervals 0–3, 3–6, 6–12, 12–24, and 24–36 h after birth and 0–3, 3–6, 6–12, 12–24, 24–48, and 48–72 h after surgery [47, 48].	Postnatal regional cerebral oxygen saturation, postoperative regional cerebral oxygen saturation, and change between postnatal and postoperative regional cerebral oxygen saturation	Mean (and SD) or median (and IQR) per group	Postnatal (until at least 24 h after birth) Postoperative (until at least 48 h after surgery)
General movements	Assessment of general movements, using the motor optimality score [49–51].	Motor optimality score at 3 months	Mean (and SD) or median (and IQR) per group	3 months
Motor, cognitive, and speech/language development	Motor, cognitive, and language composite score of the Bayley-III-NL. An average Bayley-III-NL score is 100; one SD above or below the mean concerns 15 points [52].	Motor, cognitive, and language composite score at 24 months	Mean (and SD) or median (and IQR) per group and proportion per group below 1 SD	24 months
Quality of life	Parent-reported quality of life score using the TNO-AZL Preschool Children’s Health-Related Quality of Life questionnaire [53].	Parent-reported quality of life score at 24 months	Mean (and SD) or median (and IQR) per group	24 months
Cost-effectiveness of allopurinol	Questionnaires on the need for healthcare resources, productivity losses of parents, and expenses borne by families. Healthcare and societal costs will be determined with the Dutch guidelines for health economic evaluation.	Healthcare and societal costs at 3 months; healthcare and societal costs at 24 months	Mean (and SD) per group	3 months 24 months
Cost-effectiveness of allopurinol	Length of hospitalization and intensive care stay (days) and costs associated with hospitalization using standard unit prices from Dutch guidelines for health economic evaluation.	Healthcare costs	Mean (and SD) per group	24 months
Cost-effectiveness of allopurinol	Motor, cognitive, and language composite score of the Bayley-III-NL [52] and healthcare and societal costs. Healthcare and societal costs will be determined with the Dutch guidelines for health economic evaluation.	Bayley-III-NL score, healthcare, and societal costs	Cost per point improvement in neurodevelopmental outcome per group	24 months
Pharmacokinetic evaluation of allopurinol	Allopurinol, oxypurinol, hypoxanthine, xanthine, and uric acid levels (mg/L) will be measured in blood samples collected around birth and surgery in 24 patients with a prenatal diagnosis [20, 32, 33, 54].	Allopurinol, oxypurinol, hypoxanthine, xanthine, and uric acid levels (mg/L)	Mean (and SD) or median (and IQR)	Postnatal (umbilical cord and 15–60 min, 4 h, 12 h, 13–14 h, 24 h, 36–48 h, and 96–168 h after birth) Perioperative (0–3 h before dose 3, 1–3 h after dose 3, 0–1 h before dose 4, at the end of CPB, 4 h after surgery, 0–3 h before dose 5, 1–3 h after dose 5, and 48 h after surgery)
Redox and antioxidant state of allopurinol	Enzyme-linked immunosorbent assay will be used to measure hypoxic tissue injury, overall redox and antioxidant state, lipid peroxidation, protein oxidation, and nitrosative state. Mass spectrometry will be used to determine allopurinol, oxypurinol, non-protein bound iron, and xanthine oxidase levels (mg/L) [12].	Hypoxic tissue injury, overall redox and antioxidant state, lipid peroxidation, protein oxidation, and nitrosative state Allopurinol, oxypurinol, non-protein bound iron, and xanthine oxidase levels (mg/L)	Mean (and SD) or median (and IQR) per group	Postnatal (umbilical cord) Perioperative (directly before surgery and 6 h, 1 day, 2 days, and 4 days after surgery)

*Abbreviations*: *aEEG* Amplitude-integrated electroencephalography, *Bayley-III-NL* Bayley Scales of Infant and Toddler Development - Third Edition - Dutch Norms, *BMI* body mass index, *CCHD* critical congenital heart disease, *CPB* cardiopulmonary bypass, *IQR* interquartile range, *MRI* magnetic resonance imaging, *NIRS* near-infrared spectroscopy, *SD* standard deviation

### Study procedures and visits

**Fig. 2:**
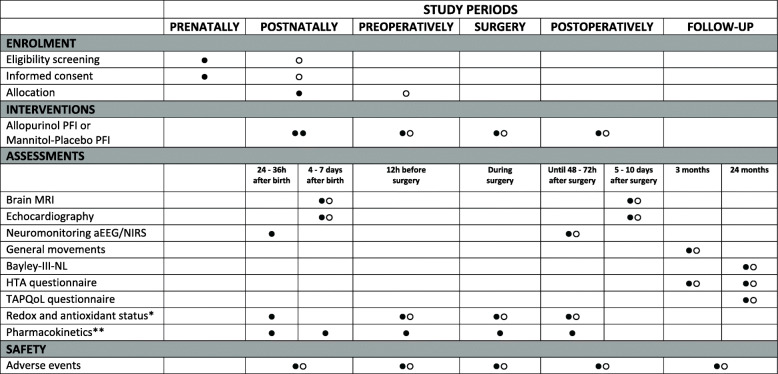
Participant timeline. Black circles (●) indicate neonates with a prenatal CCHD diagnosis. White circles (○) indicate neonates with a postnatal CCHD diagnosis. *In included subjects from the Erasmus Medical Center Rotterdam and University Medical Center Utrecht. **In the first 24 subjects with a prenatal CCHD diagnosis in UMC Utrecht. aEEG, amplitude-integrated electroencephalography; Bayley-III-NL, Bayley Scales of Infant and Toddler Development - Third Edition - Dutch Norms; CCHD, critical congenital heart disease; h, hours; HTA, health technology assessment; MRI, magnetic resonance imaging; NIRS, near-infrared spectroscopy; TAPQoL, TNO-AZL preschool children’s health-related quality of life

An overview of consecutive study procedures and visits for participants is listed in Fig. 2.

#### Brain MRI

Brain MRI will be performed pre- and postoperatively on a 1.5- or 3.0-T MRI scanner to assess the presence, severity, and volume of ischemic and hemorrhagic parenchymal brain lesions. The MRI scanning protocol includes at least T1-, T2-, diffusion-, and susceptibility-weighted imaging and MR venography [8, 39, 41]. Preoperative MRI will take place within 4–7 days after birth or at least preoperatively. Postoperative MRI will take place preferably within 5–10 days after surgery or at least within 1 month following CPB.

#### Echocardiography

Echocardiography will also be executed pre- and postoperatively, preferably on the same day as the brain MRI, to evaluate potential cardioprotective effects of allopurinol [57]. The echocardiography protocol emphasizes on cardiac systolic and diastolic function, including global ventricular function, ejection (Simpson’s biplane method or four-dimensional) and shortening fraction, ventricular dimensions, velocity over the cardiac valves (pulsed/continuous wave Doppler), cardiac valve insufficiency, longitudinal strain and tissue Doppler imaging (myocardial deformation indices), and systolic and diastolic pulmonic vein flow [44].

#### Neuromonitoring

Brain function will be assessed by amplitude-integrated electroencephalography (aEEG) and oxygenation by near-infrared spectroscopy (NIRS) at defined time points after birth (0–3, 3–6, 6–12, 12–24, 24–36 h) and surgery (0–3, 3–6, 6–12, 12–24, 24–48, 48–72 h) [45, 47]. Dominant background pattern and changes in background pattern over time (continuous normal voltage, discontinuous normal voltage, burst suppression, continuous low voltage, flat trace) and presence of sleep-wake cycling and seizure activity (absent, single seizure, repetitive seizures, status epilepticus, including response to anti-epileptic drugs) will be identified with aEEG [45]. The mean regional cerebral oxygen saturation (rScO_2_), lowest rScO_2_ (%), and duration of rScO_2_ < 45% will be assessed by NIRS [47]. Neuromonitoring will be performed after birth with continuation for at least 24 h, and after surgery with continuation for at least 48 h.

#### Neurodevelopmental follow-up

At 3 months, general movements will be systematically recorded and assessed, including a detailed score of the entire motor repertoire, using the motor optimality score [49, 50]. At 24 months, motor, cognitive, and language development will be scored with the Bayley Scales of Infant and Toddler Development - Third Edition, Dutch Norms (Bayley-III-NL), and neurological examination to objectify possible cerebral palsy is performed. An average Bayley-III-NL score is 100, one standard deviation above or below the mean concerns 15 points [52]. Children with an underlying syndromic disorder will be analyzed separately, as this group is expected to score lower on neurodevelopmental outcomes [58].

#### Cost measurement

The use of healthcare resources, productivity losses of parents, and expenses borne by families will be collected with questionnaires among parents during follow-up visits at 3 and 24 months. In addition, we will monitor the length of intensive care stay and hospitalization during the trial and estimate the costs associated with hospitalization using standard unit prices from the Dutch guidelines for health economic evaluation.

#### Quality of life

The TNO-AZL Preschool Children’s Health-Related Quality of Life Questionnaire for parents is a systematic, valid, and reliable tool that will be used to score the quality of life of CCHD infants at the age of 24 months [53].

#### Pharmacokinetics of allopurinol

In the first 24 neonates with a prenatal CCHD diagnosis in the UMC Utrecht, blood samples will be collected postnatally and perioperatively for pharmacokinetic evaluation of allopurinol. It is expected—depending on the randomization—that approximately 12 of these neonates received allopurinol PFI and 12 mannitol-placebo PFI. This analysis will be performed to extend knowledge on the current pharmacokinetic data of allopurinol, oxypurinol, hypoxanthine, xanthine, and uric acid and to establish if expected target concentrations of allopurinol > 2 mg/L are reached in CCHD neonates [54]. Blood sampling will be combined with clinically indicated samples and will be taken from pre-existent arterial or venous access lines.

#### Redox state

In order to quantify and assess the antioxidant properties of allopurinol after ischemia-reperfusion events and correlate redox biology to ischemia-reperfusion status, additional blood and urine samples of included subjects from the Erasmus MC Rotterdam and UMC Utrecht will be collected at fixed intervals (umbilical cord, preoperatively, 6 h postoperatively, and on days 1, 2, and 4 postoperatively). Using enzyme-linked immunosorbent assay, ROS analyses will be performed, including hypoxic tissue injury, overall redox and antioxidant state, lipid peroxidation, protein oxidation, and nitrosative state. Mass spectrometry will be used to determine allopurinol, oxypurinol, non-protein bound iron, and XO levels. The resultant molecular framework will provide a redox biological basis for allopurinol suppletion and couple the restoration of the redox imbalance to cerebral hypoxia-ischemia.

### Statistical analysis

#### Primary analysis

The primary analysis will be the *Z*-test for the difference of proportions between the allopurinol group and mannitol-placebo group on the primary composite endpoint. This primary analysis will be performed on the total study population and the subgroup of neonates with a prenatal diagnosis of CCHD, where significance for the prenatal group is only declared if the overall result has been demonstrated.

#### Interim analysis

One interim analysis, with the option to stop for efficacy or futility, is planned after outcomes are available for 50% of the patients. Stopping for efficacy at the interim analysis is based on the O’Brien-Fleming type alpha-spending function, which corresponds to a significance level of 0.0054 (two-sided) if the interim analysis is at exactly 50% of the planned sample size. Early stopping is only considered when the O’Brien-Fleming criteria are met for both the analysis on the full population and the subgroup with prenatal diagnosis. Statistical testing at completion will follow the same hierarchy, at the O’Brien-Fleming adjusted two-sided alpha level. The overall type I error remains controlled at the 5% two-sided, in view of the hierarchical procedure. An independent statistician and a data monitoring committee (DMC) have access to the interim results. The DMC will advise the principal investigator on the continuation of the trial. The principal investigator is responsible for deciding to continue, modify, or terminate the trial.

#### Secondary analysis

Secondary analysis is based on the comparisons of secondary outcomes between the allopurinol and mannitol-placebo group, both for the total study population and separately for subgroups of CCHD. Additionally, a secondary analysis will be performed to assess an interaction with gender, as females possibly benefit more from allopurinol administration than males [30]. Descriptive statistics will be applied, including the independent sample *t*-test or the Mann-Whitney *U*-test for continuous data and Pearson’s chi-square test for categorical data. Safety analyses will be based on incidences of (serious) adverse events in both groups.

#### Cost-effectiveness analysis

The mean costs from a healthcare and societal perspective will be calculated for both treatment groups over a 24-month observation period. Cost differences between both groups will be related to the differences in neurodevelopment, as observed with the Bayley-III-NL. Incremental cost-effectiveness will be expressed as cost per point improvement in neurodevelopmental outcomes. Probabilistic sensitivity analysis will be done with bias-corrected bootstrap analysis with 5000 samples. Cost-effectiveness planes and acceptability curves will be plotted.

#### Sample size

The sample size is calculated based on an assumed reduction in (absolute) incidence of 20% in the more prevalent subgroup of prenatal diagnosis (± 80% of the total population, control event rate assumed 71%) and a reduction of 9% in the postnatal diagnosis group (± 20% of the population, control event rate assumed 55%). With a total sample size of 236 CCHD neonates, of which 188 in the prenatal subgroup and 48 in the postnatal subgroup, the study has approximately 80% power to demonstrate the advantage of allopurinol in the total group with a two-sided alpha of 5%. The power of the hierarchical testing strategy is only slightly affected, given the large proportion of the prenatal group (80%). The effect sizes are a relevant and viable expectation and are based on the results of previous neuroprotective studies on head cooling in neonatal encephalopathy and perioperative allopurinol administration in CCHD neonates [26, 59]. RStudio version 3.6.1 was used for the sample size analysis.

Participants will be substituted in the following cases: (1) when cardiac surgery with CPB in the first month of life is unexpectedly not required or (2) when there is consent withdrawal before postoperative MRI. It is to be expected that these cases rarely occur.

#### Statistical methods to handle missing data

All randomized participants will be included in an intention-to-treat analysis, i.e., participants who prematurely discontinue the study intervention will be analyzed according to the group they were initially allocated [60]. Missing data will be handled with multiple imputation.

#### Study duration

It is estimated that approximately 115 CCHD neonates will be eligible for the study in the participating centers each year. Based on a previous neuroprotective trial and pilot study in our center, we expect an inclusion rate of approximately 80% [61]. A duration of 5 years, including an inclusion period of 3 years (corresponding to 80 CCHD neonates per year and an inclusion rate of 70%) and a 2-year follow-up, is expected to be feasible for study completion.

### Data management and monitoring

Senior data managers from the UMC Utrecht (sponsor) will supervise and assist data management during the trial. A data management system (Castor Electronic Data Capture) will be used to enter relevant data of each participant in electronic case report forms (eCRFs). These forms are created by the study team and include items on baseline, study medication, imaging, adverse events, clinical characteristics, and all primary and secondary outcomes. In the eCRFs, the participants will be pseudonymized and presented with their study identification number and allocation number. Castor Electronic Data Capture complies fully with Good Clinical Practice guidelines; the data will be traceable from logging in to data modification. The electronic database is secured through role-based access: only the study team will have access to the database, after permission from the principal investigator. Complex validation rules in the CRF are available to ensure that the collected data is complete, consistent, and correct. Additionally, we built in range checks for data values to promote data quality. Source data will be stored securely in each participating center. The imaging data will be shared via the Research Digital Imaging and Communications in Medicine server of the sponsor. An independent monitor will perform routine inspections at each study site to secure study integrity and quality of data. The case report forms and futher details on data management procedures are available upon request from the corresponding author.

### Safety

An independent DMC with extensive clinical research experience will evaluate the safety and efficacy data at regular intervals and advise on the results of the planned interim analysis. This DMC consists of a pediatric neurologist, pediatric intensivist, pediatric cardiologist, and a clinical epidemiologist. The study team will screen the medical records of participants daily to identify any adverse events. In addition, we will instruct the parents/legal guardians to contact the study team if adverse events occur during the follow-up period of 24 months. The nature, severity, and causal relationship with the study medication of each adverse event occurring during the trial will be evaluated. All details, such as duration, clinical symptoms, treatment, and outcome of the event, are recorded by the study team on case report forms. The Medical Dictionary for Regulatory Activities Terminology will be used for the coding of adverse events. Unexpected serious adverse events will be reported to the Medical Research Ethical Committee and DMC. It is known that CCHD and cardiac surgery itself can result in several adverse events. Therefore, expected disease-related events will be recorded on separate case report forms, which are presented in Additional file 2. The safety analysis will include all CCHD neonates, including subjects who withdraw from participation during the study. However, we will only collect their safety data from the period that they participated in the study. In trial publications, we will include all unexpected and expected adverse events that are significantly more present in the allopurinol group compared to the mannitol-placebo group. Further details are described in a DMC charter, which is available upon request from the corresponding author.

### Blinding

The study team, care providers, parents, and assessors of neurodevelopmental outcomes are blinded to the allocation in the trial (quadruple blinding). Blinding of the study team will be maintained throughout the inclusion period and until all CCHD neonates underwent primary outcome assessment. This can be secured as allopurinol PFI has an identical appearance as mannitol-placebo PFI. Blinding of parents, care providers, and assessors of neurodevelopmental outcomes will be continued until all follow-up visits at 24 months are completed. The following parties are unblinded during the trial: (1) Ace Pharmaceuticals, for randomization, preparation, and labeling of study medication; (2) UMC Utrecht Pharmacy, operating as a central distributor of study medication to all study sites; (3) an independent laboratory, to analyze the pharmacokinetic data; and (4) the DMC, if it requests full unblinding to arrive at a recommendation. An independent statistician will prepare the DMC reports and planned interim analysis by group level (A vs. B) and will not be aware of the actual treatment assignment. Premature unblinding by opening an “emergency breaking code envelope” of a specific CCHD neonate may take place when knowledge of the study medication arm is necessary for adequate treatment of a (serious) adverse event.

### Ethical considerations

#### Regulatory aspects

The trial (EudraCT 2017-004596-31; ClinicalTrials.gov NCT04217421) will be conducted according to the principles of the Helsinki Declaration (2013). The trial is approved by the Medical Research Ethical Committee of the UMC Utrecht (reference number NL62772.041.18) and the Dutch competent authority “Centrale Commissie Mensgebonden Onderzoek” (reference number NL62772.041.18). Protocol modifications will be submitted to these trial regulators for approval and will be communicated with all relevant parties, e.g., participants, study sites, and trial registries.

#### Recruitment and consent

Parents of neonates with a prenatal diagnosis of CCHD are informed about the study by the obstetrician and/or fetal cardiologist between 24 and 36 weeks of gestation and are asked for written consent before the start of delivery. Parents of neonates with a postnatal diagnosis of CCHD are informed about the trial by the pediatric cardiologist shortly after the diagnosis and have to give written consent at least 24 h before cardiac surgery. The information letter and consent form are provided in Additional file 3.

#### Risk and benefit assessment

The investigational medicinal products will be studied in neonates with CCHD at high risk of brain injury. CCHD neonates are vulnerable patients who are unable to give consent themselves and are potentially at risk for drug interactions and adverse events of allopurinol. However, the need for pharmacological neuroprotection strategies in this neonatal CCHD population is evident. Previous data suggesting neuro- and cardioprotective effects of neonatal allopurinol doses of 20 mg/kg with low risk of severe side effects should be verified in a large, well-designed trial to prove efficacy or futility. As this trial is placebo-controlled, half of the CCHD neonates will not have a potential beneficial effect of allopurinol. Nevertheless, the use of a placebo agent seems justifiable as study participants often benefit from participating in a randomized controlled trial, considering the potential benefit for future CCHD neonates, and the fact that mannitol-placebo PFI 20 mg/kg is safe [38, 62]. Care is taken to ensure that the study-driven burden for all participants is kept to a minimum.

#### Provisions for post-trial care

Post-trial care is deemed not to be necessary for this trial. Participants will not receive compensation for trial participation. The sponsor has an insurance, following legal requirements for clinical research in the Netherlands, that provides cover for damage to research subjects through injury or death caused by study procedures. The insurance applies to the damage that occurs during the trial or within 4 years after trial termination.

### Results and dissemination

The final trial results will be posted on the registration page of ClinicalTrials.gov and presented via publication in scientific journals. Additionally, the results will be communicated to the parents or legal guardians of study participants. An individual or group that substantially contributed to the conception, design, acquisition, analysis, or interpretation of the trial will be granted authorship, provided that they also contribute to the drafting of the manuscript or critically revise the manuscript. We do not intend to use professional writers.

#### *Ancillary studies*

On the consent form, parents or legal guardians are asked for permission to share participant data and maternal data within the European Association Brain in Congenital Heart Disease (EU-ABC) research consortium that currently consists of the UMC Utrecht, University Children’s Hospital Zurich, University Hospital Giessen, German Heart Center Munich, and King’s College Hospital London. Future research using the data generated in this trial might include studies investigating the effect of allopurinol on neurodevelopmental outcomes after 24 months.

## Discussion

The CRUCIAL trial focuses on the neuro- and cardioprotective effects of allopurinol administration early after birth and around cardiac surgery with CPB in neonates with CCHD. We aim to provide definite answers on allopurinol’s immediate effects on brain injury and cardiac function, as well as late effects on neurodevelopment. If allopurinol proves to be beneficial in limiting parenchymal brain injury, our subsequent goal is to make allopurinol clinically available for all CCHD neonates.

Large randomized controlled trials that include both structural brain imaging with MRI and long-term follow-up are needed to investigate the effects of neuroprotective drugs in CCHD neonates. A phase III, randomized, quadruple-blinded, placebo-controlled, multicenter study is the most adequate design to prove the efficacy or futility of allopurinol as a neuroprotective agent in the CCHD population. A phase III study seems justifiable since previous studies have already suggested neurocardiac protective effects of allopurinol without serious side effects [24–26]. Multiple pediatric heart centers should be involved to include our anticipated sample size of CCHD neonates considering the relatively low prevalence of CCHD and the heterogeneity of cardiac defects [1]. An interim analysis halfway with the option to stop for efficacy or futility ensures that allopurinol is not unethically withheld or unnecessary given to subsequent CCHD neonates [61].

Today in the Netherlands, most neonates are diagnosed with CCHD before birth (approximately 80%) due to improvements in prenatal diagnostics over the past years [8]. As the proportion of CCHD neonates with a prenatal diagnosis is expected to further increase in the following years, this is the primary subgroup of interest in our trial. This group will receive allopurinol or mannitol-placebo early postnatally and perioperatively [63]. As the subgroup of CCHD neonates with a postnatal diagnosis (approximately 20%) can be considered to be at even higher risk for brain lesions (as they often are admitted to the intensive care unit with respiratory and circulatory insufficiency), these patients will also be included in this trial, although study medication can only be given perioperatively in this group [34]. The total group (*n* = 236) and subgroup of CCHD neonates with a prenatal diagnosis (*n* = 188) will be primarily analyzed and the postnatal diagnosis subgroup (*n* = 48) secondarily. A beneficial effect of allopurinol in both the total and prenatal group will be required to apply for market registration and to make allopurinol available for all CCHD neonates, including those with a postnatal diagnosis.

The brain of neonates with CCHD is at high risk for parenchymal injury, especially ischemic lesions such as white matter injury and stroke [8]. Since we now know that brain injury mainly occurs in the vulnerable periods early after birth and around cardiac surgery with CPB, this offers opportunities for investigating the effects of neuroprotective drugs in the CCHD population within a clear time frame [19]. By administering and reaching target levels of allopurinol before potential cerebral hypoxic-ischemic events occur, this “pre-treatment” maximizes the chance of success in reducing parenchymal brain lesions [64].

Dosage and timing of allopurinol administration (20 mg/kg) are based on previous pharmacokinetic and clinical studies performed in neonates who underwent cardiac surgery and neonates with perinatal asphyxia [26–29, 54]. XO inhibition by allopurinol, reducing the production of ROS, was reached with 5–10 mg/kg in neonates with hypoplastic left heart syndrome and with 10 mg/kg (and 20 mg/kg priming dose) in neonates who underwent extracorporeal membrane oxygenation [32, 33]. High dosages of allopurinol seem to have additional neuroprotective properties as this increases scavenging of ROS and chelation of non-protein bound iron, without increasing the risk of severe adverse effects [21, 22, 27]. Previously, 2 doses of allopurinol 20 mg/kg in a 12-h interval postnatally led to improved long-term neurodevelopmental outcomes in neonates with moderate asphyxia [65]. However, in CCHD neonates, renal function should be taken into account as allopurinol is entirely cleared by the kidneys and acute kidney injury could temporarily occur after cardiac surgery with CPB [20]. Therefore, the postoperative dose will be administered 24 h (instead of 12 h) after surgery. Additionally, a pharmacokinetic substudy will be performed in the first 24 patients to monitor the levels of allopurinol directly after birth and around cardiac surgery with CPB.

We chose to include brain injury on postoperative MRI in the composite primary outcome instead of neurodevelopmental outcome at the age of 24 months. Firstly, neonatal MRI can be used as a “bridging biomarker” for long-term outcomes since ischemic white matter lesions are known to be associated with lower intelligence quotient and attention problems at school-age, and in case of posterior limb internal capsule involvement, also motor problems [36]. Consequently, the beneficial effects of allopurinol on parenchymal brain lesions are expected to improve long-term neurodevelopmental outcomes. Furthermore, early neurodevelopmental outcome at the age of 24 months seems less predictive for long-term outcomes, since CCHD neonates “grow into their deficits” and neurodevelopmental problems become more apparent at school-age [4]. Moreover, CCHD neonates with a syndromic disorder (approximately 15%) can be included as well by assessing brain injury postoperatively on MRI (as part of the composite primary outcome) instead of neurodevelopmental outcome at the age of 24 months. This syndromic CCHD subgroup is expected to score lower on neurodevelopmental outcomes and, therefore, will be analyzed separately [58]. Lastly, as many CCHD neonates undergo multi-staged cardiac repair through infancy, neurodevelopmental outcomes may exhibit a collective effect from multiple surgeries.

## Conclusions

Neonates with CCHD undergoing cardiac surgery with CPB are at increased risk of acquiring parenchymal brain lesions and subsequent long-term neurodevelopmental sequelae. Therefore, neuroprotective interventions are needed to improve the outcomes of these neonates. The CRUCIAL trial will assess the neuro- and cardioprotective effects of early postnatal and perioperative allopurinol administration in CCHD neonates. If proven beneficial and safe, allopurinol could become the first standard neuroprotective pharmacological treatment strategy in CCHD neonates.

## Trial status

Recruitment has started in January 2020. The first patient was enrolled on February 14, 2020. The recruitment is expected to be completed in January 2023, and the follow-up in January 2025. The final protocol version is version 2.1, February 2020.

## Supplementary Information

**Additional file 1.** SPIRIT Checklist.

**Additional file 2.** List of expected disease-related adverse events.

**Additional file 3.** Information letter and consent form.

## Data Availability

The coordinating and principal trial investigators have access to the final data and materials.

## Abbreviations

ACAHA: Academic Center for Congenital Heart Disease
aEEG: Amplitude-integrated electroencephalography
Bayley-III-NL: Bayley Scales of Infant and Toddler Development - Third Edition - Dutch Norms
CHD: Congenital heart disease
CCHD: Critical congenital heart disease
CPB: Cardiopulmonary bypass
DMC: Data Monitoring Committee
eCRF: Electronic case report form
MC: Medical Center
MRI: Magnetic resonance imaging
NIRS: Near-infrared spectroscopy
PFI: Powder for infusion
RNS: Reactive nitrogen species
ROS: Reactive oxygen species
UMC: University Medical Center
WFI: Water for infusion
XO: Xanthine oxidase
