# Supplementary material for: CeRebrUm and CardIac Protection with ALlopurinol in Neonates with Critical Congenital Heart Disease Requiring Cardiac Surgery with Cardiopulmonary Bypass (CRUCIAL): study protocol of a phase III, randomized, quadruple-blinded, placebo-controlled, Dutch multicenter trial
Source: Trials. 2022 Feb 23;23:174. doi: 10.1186/s13063-022-06098-y (PMC8867620; doi:10.1186/s13063-022-06098-y)
Supplement: Supplementary file 1 — Additional file 1. SPIRIT Checklist. [file 13063_2022_6098_MOESM1_ESM.doc]

**Additional file 1:**

**Standard Protocol Items: Recommendations for Interventional Trials (SPIRIT).**

Table 1: SPIRIT 2013 Checklist

| Section/item | Item number | Description on page |
| --- | --- | --- |
| **Administrative information** | | |
| Title | 1 | 1, 6 |
| Trial registration | 2a | 5, 6 |
|  | 2b | 6 |
| Protocol version | 3 | 6, 28 |
| Funding | 4 | 6, 29 |
| Roles and responsibilities | 5a | 1, 2, 6, 7 |
|  | 5b | 8 |
|  | 5c | 8, 29 |
|  | 5d | 21, 22 |
| Introduction |  |  |
| Background and rationale | 6a | 9 – 11 |
|  | 6b | 13, 22 |
| Objectives | 7 | 11 |
| Trial design | 8 | 11 |
| Methods: Participants, interventions, and outcomes | | |
| Study setting | 9 | 11, 12 |
| Eligibility criteria | 10 | 12 |
| Interventions | 11a | 13, 14, figure 1 |
|  | 11b | 14 |
|  | 11c | 14 |
|  | 11d | 14 |
| Outcomes | 12 | 14, 15, 40-42 |
| Participant timeline | 13 | Figure 2 |
| Sample size | 14 | 20 |
| Recruitment | 15 | 23 |
| **Methods: Assignment of interventions (for controlled trials)** | | |
| Allocation: |  |  |
| Sequence generation | 16a | 13 |
| Allocation concealment mechanism | 16b | 13, 14, 22, 23 |
| Implementation | 16c | 13, 14 |
| Blinding (masking) | 17a | 22, 23 |
|  | 17b | 22, 23 |
| **Methods: Data collection, management, and analysis** | | |
| Data collection methods | 18a | 15 – 18, 21 |
|  | 18b | 14, 22 |
| Data management | 19 | 21 |
| Statistical methods | 20a | 18 – 20 |
|  | 20b | 19 |
|  | 20c | 20 |
| **Methods: Monitoring** | | |
| Data monitoring | 21a | 21, 22 |
|  | 21b | 18, 19 |
| Harms | 22 | 21, 22 |
| Auditing | 23 | 14, 21 |
| Ethics and dissemination | | |
| Research ethics approval | 24 | 23 |
| Protocol amendments | 25 | 23 |
| Consent or assent | 26a | 23 |
|  | 26b | 24, 25 |
| Confidentiality | 27 | 21 |
| Declaration of interests | 28 | 29 |
| Access to data | 29 | 29 |
| Ancillary and post-trial care | 30 | 24, 25 |
| Dissemination policy | 31a | 24 |
|  | 31b | 24 |
|  | 31c | 29 |
| Appendices |  |  |
| Informed consent materials | 32 | Additional file 3 |
| Biological specimens | 33 | 17, 18 |

*It is strongly recommended that this checklist be read in conjunction with the SPIRIT 2013 Explanation & Elaboration for important clarification on the items. Amendments to the protocol should be tracked and dated. The SPIRIT checklist is copyrighted by the SPIRIT Group under the Creative Commons “[Attribution-NonCommercial-NoDerivs 3.0 Unported](http://www.creativecommons.org/licenses/by-nc-nd/3.0/)” license.
