# Supplementary material for: CeRebrUm and CardIac Protection with ALlopurinol in Neonates with Critical Congenital Heart Disease Requiring Cardiac Surgery with Cardiopulmonary Bypass (CRUCIAL): study protocol of a phase III, randomized, quadruple-blinded, placebo-controlled, Dutch multicenter trial
Source: Trials. 2022 Feb 23;23:174. doi: 10.1186/s13063-022-06098-y (PMC8867620; doi:10.1186/s13063-022-06098-y)
Supplement: Supplementary file 2 — Additional file 2. List of expected disease-related adverse events. [file 13063_2022_6098_MOESM2_ESM.docx]

**Additional file 2: Disease related adverse events.**

In neonates with critical congenital heart disease (CCHD) requiring cardiac surgery with cardiopulmonary bypass (CPB), several complications can be expected in the clinical course after birth and around cardiac surgery. Because these complications have previously been related to the health status of CCHD or to cardiac surgery with CPB, presentation of these complications or Adverse Events are not believed to be directly related to administration of the study medication. These Adverse Events are summarized in table 2 below.

**Table 2.** *Disease related adverse events*

| **Classification** | **Disease Related Adverse Events** |
| --- | --- |
| Blood- and Lymphatic Disorders | Anemia (including need for erythrocyte concentrate transfusion)  Clotting disorders:   - Increased INR - Prolonged PTT (including need for fresh frozen plasma)   Leukocytosis  Leukocytopenia  Thrombocytopenia (including need for thrombocyte concentrate transfusion) |
| Cardiac Disorders | Circulatory failure, including:   - Arterial hypotension - Cardiac arrhythmias - Circulatory arrest - Heart block (including transient or permanent need for pacemaker implantation) - Increased concentration of CK, CK-MB, BNP, troponin - Low cardiac output syndrome - Need for hydrocortisone, inotropes, vasopressors, and volume expansion |
| Endocrine Disorders | Abnormal thyroid function tests  Hypothyroidism |
| Gastrointestinal Disorders | Emesis  Feeding problems  Lower gastrointestinal hemorrhage, including:   - Blood in stool - Hemorrhagic or necrotizing (entero)colitis   Upper gastrointestinal hemorrhage, including:   - Blood or hematin in gastric aspirates - Hematemesis - Suspected peptic ulcer |
| General Disorders | Fever  Known adverse reactions to concomitant medication  Known adverse reactions to devices (catheters, tubes, procedures), including:   - Thrombosis - Infections   Multi-organ dysfunction |
| Hearing Disorders | Abnormal acoustic stimulation tests, including:   - Abnormal BERA - Abnormal otoacoustic emissions - Deafness (excluding pre-existing congenital hearing disorders) |
| Hepatobiliary Disorders | Hepatic failure, including:   - Decreased total protein - Hypoalbuminemia - Impaired clotting - Increased direct bilirubin (cholestasis) - Increased indirect bilirubin (hyperbilirubinemia) - Increased liver enzymes - Reduced cholinesterase activity   Portal vein thrombosis |
| Infectious Disorders | Pulmonary infections:   - Pneumonia - Pneumonia, associated with (non)invasive airway support   Systemic bacterial infections:   - Suspected sepsis of infectious origin, not catheter-related - Confirmed sepsis / blood culture-proven sepsis, not catheter-related - Suspected sepsis, catheter-related - Confirmed sepsis, catheter-related |
| Immunologic Disorders | Erythema toxicum neonatorum  Fever  Sepsis of non-infectious origin |
| Metabolic Disorders | Elevated concentrations of ALT, AST, CK, or LDH  Hyperglycemia or hypoglycemia  Hypocalcemia  Persisting (lactic) acidosis |
| Nervous System Disorders | Abnormal electric brain activity  Abnormalities of feeding (insufficient sucking), muscle tone, reflexes, spontaneous movements, or vigilance  Abnormal findings on cerebral ultrasound or abnormal findings on cerebral MRI, including:   - Cerebral arterial infarction - Cerebellar bleeding - Cerebral edema - Cerebral ischemia - Cerebral sinovenous thrombosis - Intracerebral bleeding - Intraventricular bleeding - Subarachnoid bleeding - Thalamic bleeding   Seizures (including need for anticonvulsive therapy) |
| Procedural Complications Related to Neonatal Cardiac Surgery with Cardiopulmonary Bypass | Aspiration  Atelectase  Blood loss  Bronchial intubation  Bronchospasm  Cardiac arrhythmias  Cardiac tamponade  Chylothorax  Circulatory arrest  Death  Dislocation endotracheal tube  Haematothorax  Heart block (including need for transient or permanent pacemaker implantation)  Hypoxemia  Low cardiac output syndrome  Need for postoperative mechanical circulatory support (ECMO)  Need for renal replacement therapy (filtration or dialysis)  Neurological deficit  Open sternum, delayed closure of sternum  Paralyzed diaphragm, paralyzed recurrent laryngeal nerve  Post-pericardiotomy syndrome  (Pulmonary) hypertension  Refixation of the sternum  Rehospitalization  Reintubation  Renal failure  Reoperation  Pneumothorax  Seizure  Shunt obstruction  Stroke  Wound infection |
| Renal and Urinary Disorders | Electrolyte imbalance, including:   - Hypercalcemia or hypocalcemia - Hyperkalemia or hypokalemia - Hypernatremia or hyponatremia - Hyperphosphatemia or hypophosphatemia   Renal failure, including:   - Increased serum creatinine, or serum urea - Need for diuretics - Need for renal replacement therapy (filtration or dialysis) - Oliguria, anuria, polyuria   Renal tubular dysfunction, including metabolic acidosis of renal origin  Renal vein thrombosis |
| Reproductive System Disorders | Hydrocele  Maldescensus testis  Ovarial cyst |
| Respiratory, Thoracic, and Mediastinal Disorders | Apnea  Atelectase  Hypopnea  Need for inhalative nitric oxide therapy  Need for sildenafil  Pneumomediastinum  Pneumopericardium  Pneumothorax  Pneumonia  PPHN, including:   - Increased pulmonary vascular resistance - Pulmonary hypertension   Respiratory failure including:   - Diaphragm paralysis - Hypercapnia - Hypoxemia - Need for CPAP - Need for invasive or non-invasive positive pressure ventilation - Upper airway obstruction - Vocal cord dysfunction |
| Skin and Subcutaneous Disorders | Erythema toxicum neonatorum  Subcutaneous fat necrosis |
| Surgical & Medical Procedures | Inguinal hernia repair |
| Vascular Disorders | Infarctions   - Cerebral artery infarctions   Thrombosis:   - Renal vein thrombosis - Sinus vein thrombosis - Portal vein thrombosis - Vascular line related thrombosis |

*Abbreviations:* ALT: Alanine Amino Transferase; AST: Aspartate Amino Transferase; BERA: Brainstem Evoked Response Audiometry; BNP: Brain Natriuretic Peptide; CK: Creatine Kinase; CPAP: Continuous Positive Airway Pressure; ECMO: Extracorporeal Membrane Oxygenation; INR: International Normalized Ratio; LDH: Lactate Dehydrogenase; PPHN: Persistent Pulmonary Hypertension of the Newborn; PTT: Partial Thromboplastin Time; SIRS: Systemic Inflammatory Response Syndrome;
