# Supplementary material for: CeRebrUm and CardIac Protection with ALlopurinol in Neonates with Critical Congenital Heart Disease Requiring Cardiac Surgery with Cardiopulmonary Bypass (CRUCIAL): study protocol of a phase III, randomized, quadruple-blinded, placebo-controlled, Dutch multicenter trial
Source: Trials. 2022 Feb 23;23:174. doi: 10.1186/s13063-022-06098-y (PMC8867620; doi:10.1186/s13063-022-06098-y)
Supplement: Supplementary file 3 — Additional file 3. Information letter and consent form. [file 13063_2022_6098_MOESM3_ESM.docx]

**Can allopurinol reduce brain injury in newborns with critical congenital heart disease?**

**- CRUCIAL -**

**A randomized, blinded, placebo-controlled, multicenter trial.**

**
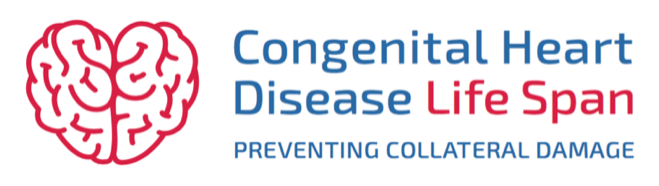

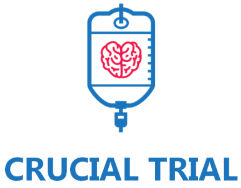
**

Dear parents and guardians,

You receive this letter because your child has been diagnosed with a critical congenital heart defect. Via this letter, we ask your permission for your child to participate in a medical-scientific study: the CRUCIAL trial. By giving the study medication allopurinol or placebo to your child, we want to examine if allopurinol can reduce possible brain injury in newborns with critical congenital heart disease.

Participation in this trial is voluntary. Written consent is necessary for the participation of your child. Before you decide whether you want your child to participate, we will give more information about the study. Please read this information carefully and ask the researcher for further information if you have any questions. It is also possible to ask the independent expert for additional information. You will find their contact details at the end of this letter. It is, of course, also possible to discuss this study with your partner, friends, or family. Additional information about participating in a study can be found in the enclosed brochure on medical-scientific research.

1. **General information about the study**

This study has been initiated by the University Medical Center (UMC) in Utrecht. The study is performed by doctors and researchers in multiple academic hospitals in the Netherlands. In total, 236 newborns with a critical congenital heart disease (diagnosed either before or after birth) will participate in this study. The Medical Research Ethical Committee has approved the study.

1. **The medical problem: Brain injury**

Newborns with critical congenital heart disease are at risk for brain injury. Brain injury can develop due to reduced oxygen delivery to the brain. This can develop shortly after birth and around cardiac surgery with cardiopulmonary bypass. Production of toxic compounds after a period of oxygen deficiency can result in brain injury. This brain injury may lead to intellectual or physical disabilities or epilepsy.

1. **Aim of the study**

We aim to evaluate whether the medication allopurinol can reduce brain injury caused by decreased blood flow or oxygen delivery to the brain shortly after birth and around cardiac surgery with cardiopulmonary bypass.

1. **The study medication: Allopurinol**

Allopurinol is a well-known drug used for treatment of gout in adults. In prior animal and small studies in newborns, allopurinol appears to reduce brain injury, improve cardiac function, and decrease the risk of long-term developmental problems and death. In newborns with critical congenital heart disease, allopurinol is not registered for prevention of brain injury and is, therefore, not standard of care.

1. **What does participation involve?**

If you decide to participate, the total study duration for your child is 24 months.

**5.1 Is your child eligible for participation?**

An ultrasound of your child’s heart was made to diagnose a heart defect. Children with a critical congenital heart disease who are expected to require an operation with cardiopulmonary bypass in the first month of their life can participate in the study. Children born prematurely, with very low birth weight, or planned to only receive comfort care, cannot participate. Your doctor decides if your child can participate and will inform you of this.

**5.2 Unexpected findings**

We may find something during the study tests that requires further medical evaluation. This is called an ‘unexpected finding’. We will always inform you of this. Further medical evaluation will be performed by your general practitioner or a medical specialist. The costs for this will have to be declared under your own insurance.

**5.3 Treatment**

We will give study medication shortly after birth and around cardiac surgery with cardiopulmonary bypass *(schematic overview of the treatment below*). Half of the children receive allopurinol, and the other half receives a placebo (an ineffective substitute). The placebo contains mannitol: this has no effect on brain injury but is used in much higher dosages to reduce brain swelling and excessive fluid. Allocation to treatment with either allopurinol or placebo is carried out automatically after birth in a random sequence. The study team, your child’s doctors, and you will not know what treatment your child received. However, when knowledge about the treatment group is necessary for adequate treatment, unblinding is possible. This will be decided by the principal investigator and coordinating investigator.

*Overview of treatment*


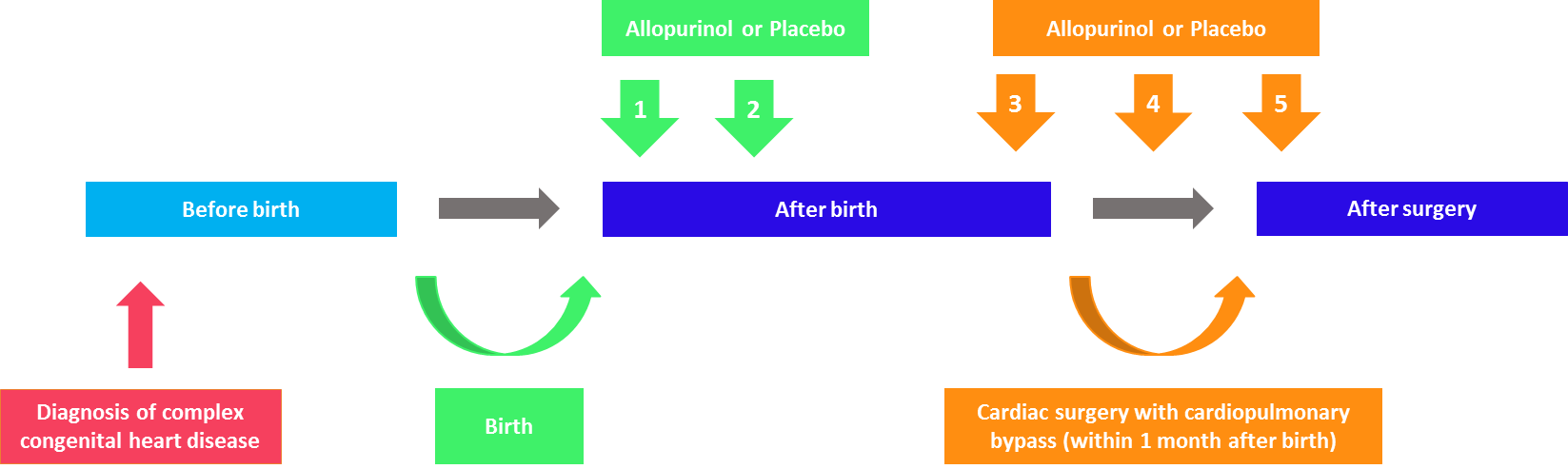


Shortly after birth *(1)* and approximately 12 hours after birth *(2),* your child will receive the study medication through intravenous administration. Approximately 12 hours before the operation *(3)*, during cardiac surgery with cardiopulmonary bypass *(4)*, and approximately 24 hours after surgery *(5)*, your child will receive the study medication through intravenous administration again.

**5.4 Standard of care**

A number of examinations of your child’s brain and heart are standard of care. The data collected in these examinations will be used to measure the effect of allopurinol. The doctor will discuss the results of these examinations with you. *The overview on page 7 shows which examinations take place and at which times.*

*Cardiac ultrasound*

An ultrasound of your child’s heart will be done on the same day as the brain MRI scan. After birth and after the operation, the function and structure of your child’s heart are examined. In this way, we can determine the effect of allopurinol on cardiac function. An ultrasound of the heart takes about 15 to 30 minutes.

*MRI scan of the brain*

Approximately 4 to 7 days after birth and 5 to 10 days after cardiac surgery, an MRI scan of the brain is performed. These scans are made to evaluate brain development and brain injury. For the CRUCIAL trial, the MRI scan after cardiac surgery is the most important as this scan is used to determine the effect of allopurinol on brain injury. This scan is made in an MRI scanner. An experienced physician will be present during this scan and will monitor your baby. The MRI scan takes about 30 minutes. Earplugs will be used to protect your child’s ears against the noise. If needed, the physician may decide to give your child medication through a nasogastric tube to aid natural sleep. This ensures that your child lies still and that the quality of the images is good.

*Assessment of brain function*

After birth (24-36 hours), before surgery (6 hours), and after surgery (48-72 hours), the following tests will be performed:

- An aEEG is a recording of brain activity to evaluate if there are any indications for epilepsy. This is measured with small electrodes on your child’s scalp.

- NIRS is used to monitor oxygen consumption of the brain with sensors placed on your child’s head.

*Neurodevelopmental assessment*

We will test the language, mental, and physical development of your child with tests and assignments. These tests are not invasive, and most children enjoy taking part. These neurodevelopmental assessments are carried out when your child is 3 months and 24 months of age.

**5.5 Additional study-driven examinations**

Some examinations are not standard care and are only performed as part of the CRUCIAL study. However, the results of these examinations will be discussed with you by the doctors. *Below and in the overview on page 7, we describe which examinations are performed in the context of the CRUCIAL study.*

*MRI scan of the heart*

The MRI scan of the heart (cardiac MRI) will be done after the MRI scan of the brain. The additional scan time for a cardiac MRI is approximately 15 minutes. The total duration of a brain MRI followed by a cardiac MRI is 45 minutes. We try to keep the burden on your child as low as possible. With cardiac MRI, we can obtain more information about the structure and function of your child’s heart and measure the blood flow and oxygen supply in the vessels that run to the brain. This provides important information on the interplay between the function of the heart and brain development and brain injury.

*Blood and urine samples*

Small amounts of blood will be drawn from your child from the umbilical cord and at various time points around cardiac surgery. These blood samples will be combined with clinically indicated blood samples or from pre-existent access lines in the blood vessels to ensure that no extra puncture is needed for study purposes. In addition, we will collect small amounts of urine at various time points around cardiac surgery. With these blood and urine samples, we measure whether allopurinol can decrease toxic compounds after a period of blood and oxygen deficiency. On the consent form, you can indicate whether you consent to these additional blood and urine samples.

*Questionnaires*

During follow-up visits at 3 and 24 months, you will receive a questionnaire. We use these questionnaires to get more insight into the healthcare costs of children with severe congenital heart disease and their parents. In addition, at 24 months, you will receive a questionnaire about the quality of life of your child and you.

1. **Possible side effects and other inconveniences**

**6.1 Possible side effects of the study medication allopurinol**

Earlier research has shown that allopurinol is safe in newborns. To date, no severe side effects occurred in any of the 374 newborns treated with allopurinol. Of these newborns, 138 received allopurinol before birth (via the mother), 58 after birth, and 178 around cardiac surgery. A possible side effect of allopurinol shown in former studies was irritation of the vessels and skin at the injection site when the intravenous catheter was not placed correctly. This might lead to scars, which occurred in a single case in a previous study. Therefore, we have set strict rules about intravenous access.

**6.2 Other possible inconveniences**

The study medication will be administered to your child intravenously. We will only use a line or intravenous catheter that is already present, meaning that we will not place new access lines just for study purposes. The burden of the additional imaging with MRI of the heart is up to 15 minutes. With MRI, no harmful radiation is used. After birth and around cardiac surgery, small amounts of blood are collected from your child. The amount of blood that will be collected is limited and will not affect the child. The questionnaires at the age of 3 and 24 months will be combined with routine follow-up visits, at the pediatric cardiologist or for neurodevelopmental assessments.

1. **Possible advantages and disadvantages**

It is important that you consider the possible pros and cons before deciding if your child can participate in the study.

**7.1 Possible advantages**

Your child has a 50% chance of receiving the study medication allopurinol. If your child is allocated to the allopurinol group, he or she might have a beneficial effect from allopurinol. This could translate into less brain damage, better function of the heart, lower risk of a physical or intellectual disability, or epilepsy. However, we do not know the exact effect of allopurinol yet; therefore, we are conducting this trial. We compare the effect of allopurinol with the effect of a placebo. If your child is allocated to the placebo group, he or she will not directly benefit from this study.

**7.2 Possible disadvantages**

Disadvantages of participating in this study are possible side effects of the study medication or possible inconveniences of the study-driven examinations. Additionally, we may find something during the study tests about your child’s health that requires further medical evaluation (an unexpected finding). You will, of course, be informed about relevant and treatable unexpected findings.

1. **What is expected of you?**

For your child’s safety and for the trial to run smoothly, it is important to make clear agreements. The agreement is that you visit us for follow-up visits when your child is 3 and 24 months. In addition, we would like you to contact the researchers when:

- You consider participation of your child in another medical-scientific study. Participation of your child in another medication study is not permitted.

- Your child starts new medication. Also when this concerns homeopathic medication, natural remedies, vitamins, or other medication from the drugstore.

- Your child is admitted or treated in a hospital.

- Your child suddenly develops health problems.

- You do not want your child to participate in the study anymore.

- Your contact details change.

1. **Resistance of your child**

Your child may resist (refuse to cooperate) during the study. The investigator will then have to stop the study immediately. In newborns, it is difficult to describe what resistance looks like. Before the start of the study you will be given an explanation of what is considered resistance. The investigator will follow the Code of Conduct on resistance of minor patients.

1. **Not participating or ending the study participation**

It is up to you to decide if your child participates in the study. Participation is voluntary. If you choose not to participate, it will not affect your child’s treatment. If your child participates, you can always change your mind and decide to stop at any time during the study. Then, only the standard of care will be continued. You do not have to explain why you want to stop participating. However, you do need to notify the investigator as soon as possible. The data collected until that moment will still be used for the study. If there is any new information about the study that is relevant to you, the investigator will contact you and you will be asked if you would like to continue with the study.

***5.4/5.5 (Continuation) Overview of examinations during the study***

**Cardiac ultrasound**

*5-10 days after surgery or at least within 1 month after surgery*

**Cardiac ultrasound**

*4-7 days after birth or before cardiac surgery*

**Allopurinol or placebo**

*12 hours before surgery*

*During surgery*

*24 hours after surgery*

**Brain function assessment**

*6 hours before surgery*

*48-72 after surgery*

**Allopurinol or placebo**

*< 45 minutes*

*12 hours after birth*

Follow-up

After the surgery

**Cardiac surgery**

*< 28 days after birth*

**Informed Consent**

*Before birth*

**Birth**

Around surgery

After birth and before surgery

Before birth

**Brain function assessment**

*24-36 hours after birth*

**Speech / language, cognitive & motor development**

**Questionnaire on healthcare utilization & quality of life**

*24 months*

**Motor development**

**Questionnaire on healthcare utilization**

*3 months*

**MRI of the brain**

*4-7 days after birth or before cardiac surgery*

*preoperatively*

**MRI of the brain**

*5-10 days after surgery or at least within 1 month after surgery*

*surgery*

1. **End of study**

The participation of your child in the study will stop if any of the following happens:

- Your child is born too early, has a low birthweight, or has a poor clinical condition;
- All study visits (mentioned in 5.4/5.5 and the overview on page 7) are completed;
- You decide to quit;
- The end of study is reached;
- The investigator decides that your child should no longer participate;
- The UMC Utrecht, government, or the Medical Research Ethical Committee decides to stop the study.

The study will end when all the participants completed the study. The study medication your child received will not be available after this study. After collecting and analyzing all data, the investigator will inform you about the main outcome of the study. The investigator can also tell you which treatment your child received (allopurinol or placebo). If you do not want to know what treatment your child received, you can inform the investigator. He/she will then not be permitted to tell you.

1. **Usage and storage of data and body material**

In this study, we collect, use, and store medical and personal data about your child and yourself (mother). Relevant data about you or your child during pregnancy will be collected from the mother’s medical records. Every child will receive a study code, which we will use for all data and body material. Your name and your child’s name will be omitted to ensure anonymity.

- 1. **The data**

All data will remain confidential. Only the research team will know which study code belongs to which child. In the written reports, only that study code will be used.

Some people are allowed to access your child’s medical and personal data. This is necessary to check whether the study is conducted correctly and reliably. This includes the following people: the research team, the Healthcare Inspectorate, a safety committee supervising the study, and a local monitor (hired by the UMC Utrecht) who checks the conduct of the study. They will keep your child’s data confidential.

After completion of the study, the data will be stored for 15 years. By signing the consent form, you give permission to collect, store, and access medical and personal data about your child. On this consent form, we also ask if we are allowed to contact you again about future research related to this study.

- 1. **Body material**

The blood and urine samples, which we use to measure whether allopurinol can decrease toxic compounds after a period of blood and oxygen deficiency, are analyzed in the Erasmus Medical Center Rotterdam. These samples will not be stored after the completion of the analysis.

**12.3 More information about your rights and your child’s rights regarding data processing**

For general information about your and your child’s rights regarding collecting and processing of the data, you can consult the website of the Dutch Data Protection Authority [www.autoriteitpersoonsgegevens.nl/](http://www.autoriteitpersoonsgegevens.nl/). You can also contact the UMC Utrecht for any questions about data collection and processing. See Appendix A for contact details.

**12.4 Future usage of data**

We would like to share the collected data with other international medical centers that we collaborate with. Only coded data will be shared. On the consent form, we ask your permission for this. In these countries, the EU law regarding the protection of personal data is not applicable. However, your privacy will be protected on a similar level as in the EU.

1. **Study registration**

A description of this clinical study can be found on <http://www.ClinicalTrials.gov>, as required by the United States’ law. This website does not contain any traceable personal information about your child. However, this website may show a summary of the study results. You have access to this website; you can find the study under protocol number NCT04217421.

1. **Insurance**

For every study participant, insurance is taken out by the UMC Utrecht. The insurance covers damage or injury caused by this study. Appendix B gives more information about this insurance.

1. **Informing the general practitioner and other medical specialists**

All medical specialists involved will be informed about your child’s participation in the study, as we will register this in the medical patient file. The general practitioner will be notified via the medical discharge letter because he/she is not involved in your child’s treatment during hospital admissions. By signing the consent form, you agree to share this information with the general practitioner.

1. **Costs/reimbursements for participating**

The study medication and study visits are free of charge. You will not be paid for your participation in this study.

1. **Registration of the consent**

During pregnancy, the participation of your unborn child will be registered in the mother's patient file. After birth, the research physician or research nurse will fill out your child's name and date of birth on the consent form and register the study participation in your child's patient file. After the delivery, you will have the opportunity to withdraw the written consent you gave before birth. Before administering the study medication, the physician responsible for your baby after birth will ask you orally if you still approve of participating in the study.

1. **Any questions?**

If you have any questions, please contact the investigator. If you would like independent advice about participation in this study, you can contact the independent expert. He or she knows a lot about the research but is not involved in the study.

If you have any complaints, please discuss this with the investigator or medical specialist. If you are unsatisfied with the conduct of the study and wish to submit a complaint, you can contact your hospital’s complaints committee.

1. **Appendices of this information letter**
2. Contact details
3. Insurance information
4. Informed Consent Form
5. Brochure: ‘Medical-scientific research: information for the study participant’.
6. **Signing the consent form**

After some time of consideration, you will be asked if you would like to participate or not. If you want to participate, we will ask you to sign the added consent form (appendix C). By signing the consent form, you confirm that you understand the information given to you and give consent for your child participating in this study. The physician will keep the signed consent form, and you will receive either a copy or a second signed consent form.

Thank you for your time.

Sincerely,

The research team of the CRUCIAL-TRIAL

**Appendix A: Contact details**

**Principal Investigator**

Name: Prof. dr. M.J.N.L. Benders

Department: Neonatology

Address: Lundlaan 6, 3584 EA, Utrecht

Phone: + 31 88 75 54 545

E-mail: m.benders@umcutrecht.nl

**Research Physician**

Name: Drs. M. (Maaike) Nijman

Department: Neonatology

Address: Lundlaan 6, 3584 EA, Utrecht

Phone: +31 6 2571 0059

E-mail: m.nijman@umcutrecht.nl

**Independent expert**

Name: Dr. M.G. Slieker

Function: Pediatric cardiologist

Address: Lundlaan 6, 3584 EA, Utrecht

Phone: +31 88 75 547 03

E-mail: m.g.slieker@umcutrecht.nl

**Complaints**

Complaints mediators

Available daily by telephone on 088 755 62 08

Website: <http://www.umcutrecht.nl/nl/Ziekenhuis/Ervaringen-van-patienten/Een-klacht-indienen>

**Data processing**

Responsible for the processing of your child’s personal data within the UMC Utrecht is the Data Protection Officer, Mr. B. van Rijn: info@umcutrecht.nl

More information can be found on the UMC Utrecht website: <https://www.umcutrecht.nl/nl/Ziekenhuis/In-het-ziekenhuis/Regels-en-rechten/Rechten>

**Appendix B: Insurance information**

The UMC Utrecht has taken out insurance for everyone participating in this study. The insurance covers damage caused by participation in this study. This includes damage during the study and up to four years after the study. Damage must be filed to the insurer within those four years.

The insurance does not cover all damage. Non-covered damage is listed briefly at the end of this text.

This is set out in the Medical Research (Human Subjects) Compulsory Insurance Decree. This decree is available on the website of the Central Committee on Research Involving Human Subjects: <https://english.ccmo.nl/> (see “Library” and then “Decrees and ministerial regulations”).

In the event of damage, please contact the insurer or insurance adjustor directly via the contact person Ms. Esther van Herk. The contact details are listed below. However, if you feel that your child has been harmed, the researcher is, of course, also willing to discuss this with you and aid you in the process.

The insurance company for this study is:

Name: CNA Insurance Company Ltd

Address: Strawinskylaan 703, 1077 XX Amsterdam

Telephone number: 020- 5737274

Policy number : 10201366

Contact person: Ms. Esther van Herk

The insurance offers a cover of €650,000 per study subject, €5,000,000 for the entire study, and €7,500,000 annually for all studies from the same sponsor.

The insurance policy does **not** cover the following damage:

- Damage as a result of a risk that you were informed about in the written information. This does not apply if the risk is more severe than expected or if the risk was very unlikely to occur;

- Damage to your health that would also have occurred if you had not participated in the study;

- Damage resulting from not or not entirely following the directions or instructions;

- Damage to descendants, resulting from an adverse effect of the study on you or your descendants;

- Damage resulting from an existing treatment method in a study into existing treatment methods.

**Appendix C: Consent Form CRUCIAL-TRIAL**

**Can allopurinol reduce brain injury in newborns with critical congenital heart disease?**

**- CRUCIAL -**

**
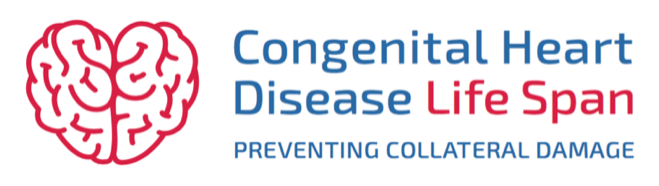
A randomized, blinded, placebo-controlled, multicenter trial.**

**
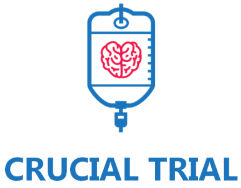
**

**Consent form for both parents or guardians**

I have been asked to give consent to let my child participate in a medical-scientific study.

To be completed after birth:

Name of study participant*:*

Date of birth:

I have read the subject information form. I was able to ask questions. My questions have been answered satisfactorily. I had enough time to decide if my child participates.

I know that participation is entirely voluntary. I know that I can decide at any moment that my child does not participate after all. I do not need to give a reason for this.

I give permission for my child’s general practitioner to be informed about participation in this study.

I give permission for my child’s treating physicians to be informed about participation in this study.

I know that some people can access my data (read: mother) and my child’s data. These people are listed in this information letter.

I consent to my child’s data to be used for the purposes stated in this information letter.

I consent to my data (read: mother) and my child’s data to be stored at the research location up to 15 years after completion of this study.

I know that I will be contacted for follow-up visits when my child reaches the age of 3 months and 24 months.

I DO/DO NOT* give consent to share my data (read: mother) and my child’s data for research collaboration with other international centers and for progress reports with involved patient associations.

I DO/DO NOT* give consent to be contacted again for new studies in the future.

I DO/DO NOT* want to be informed about the study’s progress and the treatment that my child received (placebo/allopurinol).

I DO/DO NOT* give consent to the additional blood and urine samples (as described on page 4).

I consent for the research physician/research nurse to fill out my child’s date of birth and name on this consent form.

I consent to my child participating in this study.

Name parent/guardian**:

Signature: Date: ___ / ___ / ___

Name parent/guardian**:

Signature: Date: ___ / ___ / ___

___________________________________________________________________________________________

I hereby declare that I have fully informed the person(s) mentioned above about this study. If information comes to light during the study that could affect the consent that the parents/guardians gave, I will inform them of this in a timely fashion.

Name investigator:

Signature: Date: ___ / ___ / ___

_________________________________________________________________________________________

Additional information was given by (if applicable):

Name:

Job title:

Signature: Date: ___ / ___ / ___

________________________________________________________________________________________

* Delete as appropriate.. ** Because your child is under the age of 18, both parents (or guardians) have to sign this consent form.

*The parents (or guardians) will receive the full information letter, together with a signed copy of the consent form.*
